# Bereaved family members’ perceptions of the quality of end-of-life care across four types of inpatient care settings

**DOI:** 10.1186/s12904-017-0237-5

**Published:** 2017-11-25

**Authors:** Kelli Stajduhar, Richard Sawatzky, S. Robin Cohen, Daren K. Heyland, Diane Allan, Darcee Bidgood, Leah Norgrove, Anne M. Gadermann

**Affiliations:** 1 School of Nursing and Institute on Aging and Lifelong Health, University of Victoria, PO Box 1700 STN CSC, Victoria, BC V8W 2Y2 Canada; 2 School of Nursing, Trinity Western University, 7600 Glover Road, Langley, BC V2Y 1Y1 Canada; 3 Oncology and Medicine, McGill University, Lady Davis Research Institute, Jewish General Hospital, 845 Sherbrooke Street West, Montreal, QC H3A 0G4 Canada; 4 Critical Care Medicine, Queen’s University, 76 Stuart Street, Kingston, ON K7L 2V7 Canada; 5 College of Nursing, University of Saskatchewan, 104 Clinic Place, Saskatoon, SASK S7N 2Z4 Canada; 6 Institute on Aging and Lifelong Health, University of Victoria, PO Box 1700 STN CSC, Victoria, BC V8W 2Y2 Canada; 7 Palliative Care, Saanich Peninsula Hospital, Island Health, 2166 Mt. Newton X Road, Saanichton, BC V8M 2B2 Canada; 8 School of Population and Public Health, University of British Columbia, 2206 East Mall, Vancouver, BC V6T 1Z3 Canada

**Keywords:** Bereaved family members’, Quality of care, Inpatient healthcare settings, End-of-life care, Palliative care

## Abstract

**Background:**

The aims of this study were to gain a better understanding of how bereaved family members perceive the quality of EOL care by comparing their satisfaction with quality of end-of-life care across four different settings and by additionally examining the extent to which demographic characteristics and psychological variables (resilience, optimism, grief) explain variation in satisfaction.

**Methods:**

A cross-sectional mail-out survey was conducted of bereaved family members of patients who had died in extended care units (*n* = 63), intensive care units (*n* = 30), medical care units (*n* = 140) and palliative care units (*n* = 155). 1254 death records were screened and 712 bereaved family caregivers were identified as eligible, of which 558 (who were initially contacted by mail and then followed up by phone) agreed to receive a questionnaire and 388 returned a completed questionnaire (response rate of 70%). Measures included satisfaction with end-of-life care (CANHELP- **Can**adian **H**ealth Care **E**va**l**uation **P**roject - family caregiver bereavement version; scores range from 0 = not at all satisfied to 5 = completely satisfied), grief (Texas Revised Inventory of Grief (TRIG)), optimism (Life Orientation Test – Revised) and resilience (The Resilience Scale). ANCOVA and multivariate linear regression were used to analyze the data.

**Results:**

Family members experienced significantly lower satisfaction in MCU (mean = 3.69) relative to other settings (means of 3.90 [MCU], 4.14 [ICU], and 4.00 [PCU]; *F* (3371) = 8.30, *p* = .000). Statistically significant differences were also observed for CANHELP subscales of “doctor and nurse care”, “illness management”, “health services” and “communication”. The regression model explained 18.9% of the variance in the CANHELP total scale, and between 11.8% and 27.8% of the variance in the subscales. Explained variance in the CANHELP total score was attributable to the setting of care and psychological characteristics of family members (44%), in particular resilience.

**Conclusion:**

Findings suggest room for improvement across all settings of care, but improving quality in acute care and palliative care should be a priority. Resiliency appears to be an important psychological characteristic in influencing how family members appraise care quality and point to possible sites for targeted intervention.

## Background

The quality of care provided to the dying and their family members has become an important health and social policy issue in Canada and in much of the western world. Despite repeated policy directives encouraging a shift in the setting for health care delivery into the home and away from institutions, the majority of Canadians will spend their final days and die in inpatient care settings [1–4]. A review of location of death across 45 countries found that 54% of deaths occur in hospital [5]. Researchers have examined the quality of end-of-life (EOL) care within inpatient care settings such as hospices [6, 7] [8], acute care [9–12], extended care [13–15], critical care [16], and palliative care units [6, 17]. Yet, few studies compare the quality of EOL care across settings from the perspective of family members and, studies that do, tend to focus on comparing inpatient and home care experiences [18] or reporting on barriers to optimal care in general [19]. Little research focuses on how dying in a particular inpatient care setting (i.e., acute care medical units, palliative care units, extended care units and intensive care units) influences family members’ perceptions of the quality of care.

The focus of this research was on the perceptions of bereaved family members because they have experienced the complete episode of care, including the time of death, have important assessments to make related to care quality, and may be more free to express dissatisfaction with care once the patient has died and they are not dependent on the health care system for care. Additionally, how family members perceive the quality of care provided at the EOL can have a profound influence on how they perceive the health care system as a whole [20, 21]. One study in Western Canada, for example, showed that dissatisfaction with acute hospital care was one of the primary reasons why family members opt to provide EOL care at home, even when they are unprepared or reluctant to do so, or when care becomes overly burdensome for them [22]. Negative perceptions about care quality can also influence how family members adjust to the loss of the person close to them [23, 24]. Research has shown that family members’ dissatisfaction with the quality of EOL care is associated with negative psychological outcomes such as prolonged and pathologic grief, depression and decreased quality of life, and, in turn, can contribute to an increase in the utilization of health care resources by bereaved family members [14, 25–28]. Some research has suggested that grief following the loss of a significant other, and both patient and family member demographic characteristics [29, 30], may play a role in influencing how individuals appraise certain events in their life but studies examining these characteristics in relation to how bereaved family members perceive care quality are sparse [31]. In addition, there is some indication that psychological traits such as resilience and optimism, may play a role in how people appraise certain life events. Resilience, defined as the ability to withstand and rebound from crisis and adversity or to transform disaster into a growth experience and move forward, is believed to consist of high levels of self-esteem, personal control, and optimism [32]. While resilience affects appraisal of stress, optimism (the belief that “good things are likely to happen”) diminishes the negative impact of life’s difficult experiences [33]. Resilience and optimism have been suggested as protective against the harmful effects of stress on mental and physical health [34–37], but these concepts have received little attention when studying family perceptions of EOL care.

The overall aim of this study was to gain a better understanding of how bereaved family members perceive the quality of EOL care based on where the patient has died. We define EOL care as care for people in decline who are deemed to be terminal or dying in the foreseeable (near) future [38]. Four care settings were compared: extended care units (ECU), intensive care units (ICU), medical care units (MCU) and palliative care units (PCU). The specific research questions guiding this study were: (1) To what extent are bereaved family members satisfied with the quality of care received at the EOL? (2) Does satisfaction with care vary across different inpatient care settings? and (3) To what extent is satisfaction with care explained by differences in care settings, patient and family member demographics, and psychological variables of family members?

## Methods

This study involved analysis of data from a cross sectional survey of bereaved family members using a consecutive sample from death records.

### Sample

Structured surveys were completed by a sample of bereaved family members who had a relative or friend die on an ECU, ICU, MCU or PCU in one health region in Western Canada in the past 3–6 months. For the purpose of this study, family member was defined as the person who had the most contact with the dying patient during the last days of life and who could comment on the quality of care provided. Additional eligibility criteria included a length of stay of more than 48 h and the family member being more than 18 years old and able to speak English. Family members were excluded if their relative or friend died because of traumatic causes such as an accident, homicide, suicide, or unexpected myocardial infarction. This exclusion criterion was necessary to ensure that the sample identified was one that would be representative of people who would typically require EOL care.

### Settings

In this particular health region three ECUs were represented in the study as they had a sufficient number of resident deaths to allow for evaluation. ECUs, often referred to as nursing homes, provide longer-term residential care for people unable to remain at home and who are diagnosed with chronic conditions including, for example, frailty and dementia. Care is provided primarily by resident care aides and licensed practical nurses under the supervision of a registered nurse. Services from allied health professionals such as social workers, occupation or physical therapists, recreational therapists, chaplaincy and pharmacy also exist. A medical director oversees medical care and is responsible for implementation of relevant policies, but ongoing medical care and monitoring is provided by the patient’s general practitioner. End-of-life care is provided by facility staff with limited or no access to specialist palliative care for complex cases.

The only two ICUs in the health region, both of which are represented in the study, provide care to critically ill patients as a result of trauma or severe exacerbations of illnesses. ICUs typically have a one-to-one registered nurse-to-patient ratio, and access to in-house internists.

The seven MCUs represented in the study provide care for acutely ill patients with a variety of illnesses (malignant and non-malignant conditions), but not requiring surgical interventions. Care in MCUs is provided by licensed practical nurses and registered nurses and the registered nurse-to-patient ratio is considerably lower than for ECU and higher than for ICU (typically one nurse for five to six patients). Services from allied health professionals (most commonly respiratory technicians, social workers, chaplains, occupational and physiotherapists, nutritionists) are also available in ICU and MCU settings depending on patient and family need. Specialist palliative care services are available on referral but access is limited because of resource constraints. In this health region, no regular palliative care consult service is available to acute care, including MCUs and ICUs, or ECUs.

Finally, the only two PCUs in the health region, both of which are represented in the study, offer a total of 27 beds. One of the PCUs has 17 beds and is the primary referral site for complex symptom issues, and has in-house specialist services including palliative care physicians, counsellors, spiritual care providers and a large volunteer base. Adjunct and more limited services are available from occupational and physical therapists, music therapists and pharmacists. Nursing care is provided by licensed practical and registered nurses and supported by a Clinical Nurse Leader. The other PCU has 10 beds, with care provided by licensed practical and registered nurses. A palliative care physician is employed for approximately 10 h per week to oversee medical care and is responsible for implementation of relevant policies. Ongoing medical care and monitoring is provided by general practitioners, depending on the severity of symptoms. There are resources from allied health professionals (i.e., social work, spiritual care/chaplains, music therapy, pharmacy, occupational and physical therapy) to support the unit that are shared with other hospital units at the site. On occasion, patients from this unit are transferred to the 17-bed PCU at the request of family or when the care situation requires more resources than this unit can provide. The nurse-to-patient ratio is typically one nurse to for four to five patients on both of these units, and the large majority of patients being cared for have a malignancy.

### Recruitment

To identify eligible bereaved family members, permission was received from the health authority to screen and access death records. Registered nurses employed by the health authority were hired as research assistants to review the charts of patients who had died and identify a contact person. Letters describing the study were then sent by the research assistants (health region employees) to each eligible contact. A follow-up phone call from the research assistant was made to determine if the contact person on the chart was most involved with the patient’s care and if so, they were invited to participate. In the cases where no one answered, up to five phone calls were made on different days of the week and at different times of the day. If the identified contact person said they were unable to comment on the quality of care, they were asked to identify an alternate who was then contacted.

### Data collection

Those agreeing to participate were mailed a questionnaire by the research assistant and asked to return it in a pre-addressed stamped envelope. However, six participants (1.5% of the sample) requested to complete the questionnaire by phone and this was accommodated. Family members of patients who received care in multiple care settings in their last month of life (because of in-patient transfers) were asked to rate the care of the setting in which their loved one died. All family members signed a letter of informed consent prior to participating. Ethical approval for the study was granted by a university-based ethics review board.

### Instruments

Satisfaction with EOL care in the last month of their relative or friend’s life was measured using the CANHELP^a^ (**Can**adian **H**ealth Care **E**va**l**uation **P**roject) instrument [39, 40]. This self-report instrument was developed and validated for patients at the EOL and their family members to assess satisfaction with a variety of actionable items. The CANHELP Bereavement version used in this study was a 43-item instrument with two items about overall satisfaction with care, and 41 items that fall into one of six quality of care subscales that each had good internal consistency reliability (based on the ordinal alpha; [41]) in our study sample: doctor and nurse characteristics (8 items; ordinal alpha = 0.94); illness management (9 items; ordinal alpha = 0.92); health service characteristics (4 items; ordinal alpha = 0.81); communication and decision-making (11 items; ordinal alpha = 0.92); you and your relationships with others (5 items; ordinal alpha = 0.73); and spirituality and meaning (4 items; ordinal alpha = 0.89). Responses to each item are scored on a 5-point scale ranging from ‘not at all satisfied’ (1) to ‘completely satisfied’ (5). A ‘not applicable’ response category is available for items that family members believe do not apply to their particular situation. The subscale scores are the means of the corresponding items and the total score is the mean of the 41 items (ordinal alpha = 0.96), with higher scores indicating greater satisfaction (ranging from 1 to 5).

In addition to demographic information collected as part of the self-report questionnaire, participant grief, optimism and resilience were also measured. Level of grief in bereavement was measured using the Texas Revised Inventory of Grief (TRIG) [42, 43]. This is a Likert-type measure in two parts. Part 1 comprises eight items, measuring initial grief around the time of death; Part 2, with 13 items, assesses present grief. Responses are scored on a 6-point scale ranging from completely true (1) to completely false (6). The authors reported acceptable internal consistency reliability (Cronbach’s alpha) of 0.77 (Part 1) and 0.86 (Part 2) and a test-rest reliability of 0.74 (Part 1) and 0.88 (Part 2). The overall TRIG score, based on the mean of all item responses, was used in the analysis. Higher scores are indicative of less grief.

Optimism was measured using the Life Orientation Test – Revised, a personality measure used to assess individual differences in generalized optimism [44, 45]. This is a 10-item measure scored on a 4-point Likert scale ranging from strongly disagree (0) to strongly agree (4). The authors provide evidence for internal consistency (Cronbach’s alpha = .78) and Given et al. [34] also reported moderate internal consistency with family caregivers of cancer patients (Cronbach’s alpha = .80). The overall optimism score based on the average of all 10 items (after reverse coding of negatively worded items) was used in this study, with higher scores indicating greater optimism.

Resilience was measured using a resilience scale developed by Wagnild and Young [46]. This measure is comprised of 25 questions using a 7-point Likert scale ranging from strong disagree (1) to strongly agree (7). Authors provide evidence for this measure’s internal consistency (Cronbach alpha = .72–.94) and test-retest reliability (*r =* .67–.84), construct validity, and concurrent validity [47]. This measure has been widely used to identify the degree of individual resilience (personal competence and acceptance of self and life) in multiple groups (adolescents, younger and older adults) [48]. The overall resilience measure, based on the average mean score of all items, was used in this study, with higher scores indicating greater resilience.

### Data analysis

Descriptive statistics (means and standard deviations [SD] for continuous variables and percentages for categorical variables) were used to descibe the demographic variables. To examine differences in demographics between settings, chi-square tests were used for the categorical variables, and analysis of variance (ANOVA) was used for age, a continuous variable. The first research question was addressed by examining the distributions of the CANHELP total and subscale scores.

Analysis of covariance (ANCOVA) was used to evaluate the extent to which the CANHELP total score and the subscale scores differed across the four types of settings (research question #2), while controlling for variability in patient characteristics (age, gender, type of cancer), caregiver characteristics (age, gender, employment status, relationship to patient, provided care, lived with care recipient), and psychological variables of family members (optimism, resilience, grief). Multivariate linear regression was used to examine the extent to which CANHELP total and subscale scores (dependent variables) were explained by the following independent variables: type of care setting, the same patient and family member characteristics as noted above, and the psychological variables of family members (optimism, resilience, grief) (research question #3). A *p*-value of <.05 was considered indicative of statistical significance. For each dependent variable, a Pratt Index was computed to evaluate the relative importance of each independent variable [49]. The Pratt Index values represent the percentages of the explained variance in the dependent variable (i.e., the R-squared) that are attributable to each independent variable in the regression analysis.

Multiple imputation, using mean and variance adjusted weighted least squares estimation, was used to create 20 imputed data files with imputed values for missing data for variables that were included in the analysis (total missing was 0.9%). In addition, based on recommendations by Holman et al. [50] multiple imputation was used to impute the “not applicable” response category for the CANHELP items (total “not applicable” was 9.0%).

## Results

In total, 712 of the 1254 patient death records screened over a 21 month period identified eligible family member participants. Of the 712 who were invited to participate, 558 agreed to have their name given to the project coordinator and were mailed the questionnaire. A total of 388 usable questionnaires were returned resulting in a response rate of 70% of surveys sent and 54% of eligible family members. Reasons for non-participation in the study from those who agreed to receive the questionnaire but did not return it included: being too busy to participate; not being interested; not wanting to re-live the experience; believing that the survey would be emotionally challenging; and believing that the majority of questions did not apply to their experience. Of the 388 family members who responded, 155 rated their satisfaction with PCUs, 140 with MCUs, 63 with ECUs, and 30 with ICUs.

### Sample description by setting

Results pertaining to the comparison of demographic characteristics for both family members and patients (care recipients) across settings is provided in Table 1. There were several significant differences in demographic characteristics of care recipients and their family members across the four types of care settings (see Table 1 for details on distributions and statistical significance). Not surprisingly, the average age of care recipients was highest in the ECU setting (84.7 years) and care recipients in the ECU setting stayed much longer in care (92% stayed more than 34 days) than in any of the other settings. The shortest length of stay was in the ICU setting, with 58.6% of care recipients staying no more than 5 days. Care recipients in the ICU setting were younger (mean = 64.5 years on average) and less likely to be a relative (33.3%), relative to the other settings. Distributions of sex and age of family members were very similar across the four settings. Family members in the ICU setting were most likely to be a spouse (60.0%) of the care recipient, while family members from an ECU setting were most likely to have been caring for a parent/in-law (65.6%). The PCU setting had the highest percentage of family members (81.8%) who indicated “yes” in response to the question “Did you provide care?”. Care recipients in the PCU setting were much more likely to have cancer (72.9%) than in any of the other settings (ranging from 7.9% in the ECU to 21.4% in the MCU).

**Table 1:** Sample description: care recipients and family members

	Total (*n* = 388)	ECU (*n* = 63)	ICU (*n* = 30)	MCU (*n* = 140)	PCU (*n* = 155)	*p*
Care recipients						
Age (years)/Mean(SD) (*n* = 384)	78.4 (14.0)	84.7^b,d^ (14.1)	64.5^a,c,d^ (10.9)	79.3^b^ (13.9)	77.5^a,b^ (12.6)	.000
Female (%) (*n* = 381)	54.3	67.2^b^	33.3^a,c,d^	54.8^b^	52.9^b^	.023
Cancer (%) (n = 388)	38.4	7.9^c,d^	3.3^c,d^	21.4^a,b,d^	72.9^a,b,c^	.000
Days on unit (%) (*n* = 379)						.000
Q1: <= 5	26.9	0.0^b,c,d^	58.6^a,c,d^	29.2^a,b^	29.8^a,b^	
Q2: 5 to <= 11	23.5	1.6^b,c,d^	24.1^a^	24.8^a^	31.1^a^	
Q3: 11 to <= 34	25.1	6.5^c,d^	13.8^c^	35.0^a,b^	25.9^a^	
Q4: >34	24.5	92.0^b,c,d^	3.4^a^	10.9^a^	13.2^a^	
Family members						
Age (years)/Mean(SD) (*n* = 383)	61.2 (12.9)	63.6 (12.5)	59.6 (12.9)	60.5 (12.5)	61.3 (13.4)	ns
Female (%) (*n* = 385)	67.8	69.4	70.0	67.4	67.1	ns
Married (%) (*n* = 385)	65.5	77.4^b,d^	53.3^a^	68.8	60.0^a^	.035
Caregiving for (%) (*n* = 383)						.000
Spouse	35.8	21.3^b,d^	60.0^a,c^	28.5^b,d^	43.2^a,c^	
Parent/in-law	49.6	65.6^b,d^	20.0^a,c,d^	53.3^b^	45.8^a,b^	
Other	14.6	13.1	20.0	18.2	11.0	
Working (%) (*n* = 381)	43.8	50.0	40.0	46.0	40.3	ns
Cared for care recipient (%) (*n* = 385)	69.7	66.1^b,d^	34.5^a,c,d^	64.9^b,d^	81.8^a,b,c^	.000
Lived with care recipient (%) (*n* = 385)	46.5	37.1^b,d^	63.3^a,c^	39.1^b,d^	53.5^a,c^	.008
Psychological variables						
Optimism possible range of 0 to 4: Mean(SD) (*n* = 375)	2.79 (0.64)	2.78 (0.61)	2.86 (0.71)	2.87 (0.62)	2.70 (0.64)	ns
Resilience possible range of 1 to 7: Mean(SD) (*n* = 379)	5.73 (0.74)	5.75 (0.69)	5.70 (0.66)	5.81 (0.61)	5.66 (0.85)	ns
Grief possible range of 1 to 6: Mean(SD) (*n* = 369)	4.64 (1.15)	4.86^a^ (1.08)	4.03^a,c^ (1.26)	4.90^b,d^ (1.03)	4.43^c^ (1.19)	.000

Note. Analyses based on non-imputed data. ^a^significant difference with ECU. ^b^significant difference with ICU. ^c^significant difference with MCU. ^d^significant difference with PCU. *P*-value is based on ANOVA for continuous variables and a chi-square test for categorical variables. *ns* not significant. *Q* quartile. *SD* standard deviation

### CANHELP item, subscale and total scores by setting

**Fig. 1:**
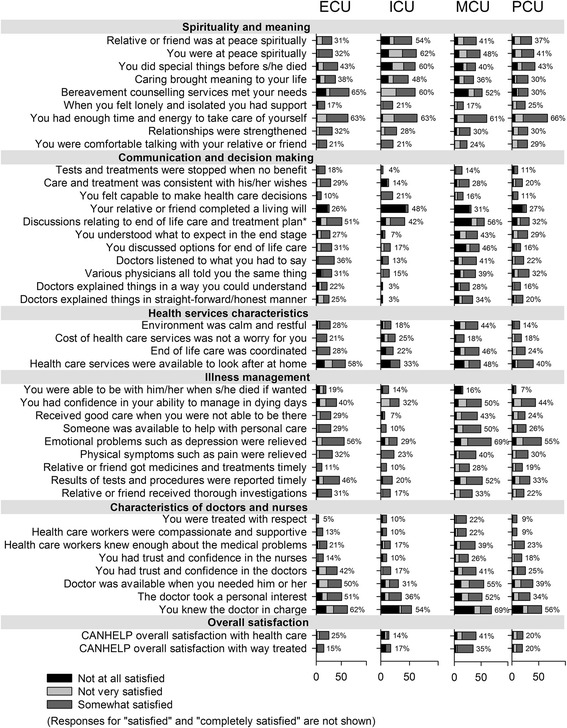
Percentages of family members within settings who are less than “satisfied” for each CANHELP item. *Note.* % refers to the percentage of people who are not “satisfied” or “completely satisfied”.* “You participated with your relative or friend in discussions with the doctor relation to his/her end of life care and treatment plan”

The relative frequencies of responses for the 43 CANHELP items are presented in Fig. 1, which reveals several observable differences between the settings and room for improvement across all settings. Family members in the MCU setting were least satisfied with overall care, with 41% reporting being less than satisfied, whereas only 14% in the ICU reported being less than satisfied. The item that had the lowest satisfaction ratings in most of the settings was “you had enough time and energy to take care of yourself” (ranging from 61% to 66% of family members who reported not being satisfied). An example of notable differences between settings includes the relatively large percentages of family members in the ECU (56%), MCU (69%) and PCU (55%) who reported not being satisfied with the relief of emotional problems of the care recipient, such as depression.

Results pertaining to the mean comparison of CANHELP total and subscale scores across settings are reported in Table 2. Family in the MCU experienced statistically significant lower satisfaction overall (CANHELP total mean = 3.68) relative to any of the other settings (means of 3.92 [ECU], 4.12 [ICU], and 4.01 [PCU]; *F*(3371) = 8.30, *p* = .000). Comparisons of subscale scores across settings reveal that satisfaction was significantly greater in the PCU than MCU for “doctor and nurse care”, “illness management”, “health services”, and “communication and decision-making”. Satisfaction in the ICU and ECU also tended to be higher than in the MCU for several of the subscales (see Table 2 for statistical significance of different subscale comparisons). There were no statistically significant differences for any of the group comparisons on the “relationships” and “spirituality and meaning” subscales. “Spirituality and meaning” was the area of least satisfaction in all settings, ranging from 3.46 in the ICU to 3.81 in the PCU.

**Table 2:** CANHELP scale and subscale means (SD) by setting adjusted for covariates

	ECU (*n* = 63)	ICU (*n* = 30)	MCU (*n* = 140)	PCU (*n* = 155)	Overall test *F*_(3371)_, *p*
CANHELP (total)	3.92(1.50)^c^	4.12(2.30)^c^	3.68(0.98)^a,b,d^	4.01(1.04)^c^	8.30, .000
Doctor and nurse care	3.87(1.97)	4.07(3.03)^c^	3.63(1.38)^b,d^	4.01(1.38)^c^	6.01, .000
Illness management	3.98(1.85)^c^	4.26(2.82)^c,d^	3.64(1.20)^a,b,d^	3.94 (1.28)^b,c^	8.39, .000
Health services	3.97(1.99)^c^	4.17(3.43)^c^	3.70(1.38)^a,b,d^	4.09(1.38)^c^	6.89, .001
Communication	3.96(1.93)^b^	4.36(2.95)^a,c^	3.74(1.26)^b,d^	4.17(1.34)^c^	9.76, .000
Relationships	3.83(1.60)	3.94(2.48)	3.80(1.08)	3.88(1.12)	2.64, .055
Spirituality and meaning	3.81(2.36)	3.46(3.68)	3.54(1.56)	3.81(1.63)	2.64, .059

*Note.* ANCOVA results for each CANHELP scale based on averages across 20 imputations. All means are adjusted for patient characteristics (age, gender, diagnosis (cancer vs. not cancer)), caregiver characteristics (age, gender, employment status, relationship to patient, provided care, lived with care recipient), and psychological variables of family members (optimism, resilience, grief).^a^statistically significant difference (*p* < .05) with ECU. ^b^statistically significant difference with ICU. ^c^statistically significant difference with MCU. ^d^statistically significant difference with PCU

### Prediction of CANHELP total and subscale scores

**Fig. 2:**
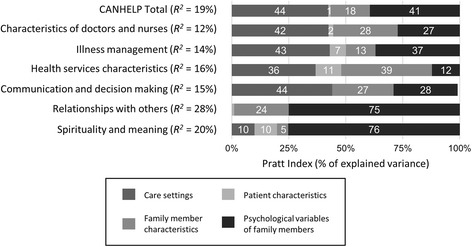
Relative importance of combined independent variables predicting CANHELP. *Note.* Pratt Index was used to compute relative importance as the percentages of explained variance attributable to variability in care settings (ECU, ICU, MCU, PCU), patient characteristics (age, gender, diagnosis (cancer vs. not cancer)), caregiver characteristics (age, gender, employment status, relationship to patient, provided care, lived with care recipient), and psychological variables of family members (optimism, resilience, grief)

The regression model including all independent variables explained 18.9% of the variance in the CANHELP total scale, and between 11.8% and 27.8% of the variance in the subscales (see Table 3). The partitioning of the explained variance is shown in Fig. 2. Most of the explained variance in the CANHELP total score was attributable to the setting of care (Pratt Index = 44%), notably receiving care in the MCU versus the PCU, and psychological characteristics of family members (Pratt Index = 41%). In particular, a one-point lower score in resilience (on a scale from 1 to 7) was associated with an average relative decrease of 0.21 in the total score (on a scale from 1 to 5). These results were similar for the “illness management” subscale, with a relative decrease of 0.18. Resilience of family caregivers also accounted for most of the explained variance in the “relationships with others” (75% of the explained variance) and “spirituality and meaning” subscales (76% of the explained variance). However, optimism was associated only with “illness management” and “spirituality and meaning” (regression coefficients of 0.15, and 0.23, respectively), and grief was associated only with “relationships with others” and “spirituality and meaning”; one-point higher score on the TRIG (i.e., less grief) was associated with a relative increase of 0.11 for both of these subscale scores. Family member characteristics (notably “employment status”), accounted for most of the explained variance in “health services characteristics” (39% of the explained variance), where family members who are employed had scores that were 0.29 points lower than those who were not employed.

**Table 3:** Multivariate regression analysis

Independent variables	CANHELP Total	Characteristics of doctors and nurses	Illness management	Health services characteristics	Communication and decision making	Relationships with others	Spirituality and meaning
Care setting (ref = palliative)							
ECU	−0.10	−0.15	0.04	−0.13	−0.21	−0.06	0.00
ICU	0.11	0.06	0.32	0.08	0.19	0.06	−0.35
MCU	−0.33*	−0.39*	−0.30*	−0.39*	−0.43*	−0.08	−0.27*
Care recipient characteristics							
Age (years)	0.00	0.00	0.00	0.00	0.00	0.00	0.01
Gender (ref = male)	0.02	0.00	−0.05	0.05	0.04	0.11	0.02
Diagnosis (cancer versus not)^a^	0.02	0.04	0.10	0.19	−0.07	0.02	−0.07
Family member characteristics							
Age (years)	0.00	0.00	0.00	0.01	0.00	0.01*	0.00
Gender (ref = male)	−0.10	−0.14	−0.15	−0.03	−0.19*	0.06	0.10
Employment status^a^	−0.12	−0.07	−0.14	−0.29*	−0.10	−0.09	−0.12
Relationship to patient							
Husband/wife (ref = ‘other’)	−0.01	−0.07	0.09	0.19	−0.01	−0.14	−0.10
Parent/parent in law (ref = ‘other’)	−0.09	−0.03	0.02	0.04	−0.22	−0.22	−0.09
Provided care^a^	0.14	0.14	0.13	0.08	0.24*	−0.02	0.09
Lived with care recipient^a^	−0.10	0.03	−0.08	0.03	−0.20	−0.31*	−0.02
Psychological variables of family members							
Optimism (possible range of 0 to 4)	0.09	0.05	0.15*	0.03	0.03	0.11	0.23*
Resilience (possible range of 1 to 7)	0.21*	0.20*	0.18*	0.14*	0.22*	0.25*	0.27*
Grief (possible range of 1 to 6)	0.01	−0.03	−0.03	0.01	0.00	0.11*	0.11*
R-square	18.90%	11.80%	14.10%	16.20%	15.30%	27.80%	19.50%

*Note.* Unstandardized regression coefficients. ^a^yes versus no (referent). *p < .05 (bolded values)

## Discussion

The overall aim of this study was to gain a better understanding of how bereaved family members perceive the quality of EOL care based on where the patient has died. While findings suggest that there is room for improvement across all settings of care, the findings particularly reveal the need for greater efforts towards improving the quality of EOL care provided in acute care medical units. This is consistent with results from other studies, which indicate that up to 35% of all hospital inpatients have palliative care needs [21, 24, 51] with these needs going largely unaddressed in acute care; patients and families report poor quality care, characterized by aggressive therapies, unnecessary pain, and depersonalized practices by providers [22, 52–56]. While resource constraints, including the absence of specialized palliative care consultation services may be one factor explaining lower overall satisfaction with acute care for bereaved family members in this study, the tendency toward curative and treatment-oriented care in acute care, and a lack of integration of palliative care approaches to care [57, 58] may also play a role. Gott et al. [59] explored how transitions to a palliative approach are managed in acute care, citing challenges such as lack of discussion with patients about prognosis and communication difficulties among team members as barriers. Indeed, in the present study, 56% of family members in acute care were not satisfied with participation in decision-making. Other research points to communication breakdowns among the interprofessional team and differing perspectives among nursing and medical staff on what constitutes quality of care for people with chronic life-limiting illnesses [60]. Additionally, a prevailing belief that dying patients “don’t belong” in acute care [60, 61] can sometimes direct providers’ attention toward a desire to discharge these patients to palliative care services instead of considering the important role that acute care medical units have in the care of the dying.

As acute care still functions as a major provider of EOL care in Canada and elsewhere [1–4], improving the quality of EOL care should be a priority, both for cancer patients - the more traditional recipients of palliative care - and for the larger population of people with life-limiting conditions such as those with advancing heart, lung and kidney disease, frailty and dementias. Integration of a palliative approach has been cited as one possible solution [57, 62, 63], but a lack of conceptual clarity about what is meant by a palliative approach hampers widespread application. Yet, a palliative approach, which involves building the capacity of providers who do not specialize in palliative care to adopt adopt the foundational principles of palliative care, adapt palliative care knowledge and expertise to the illness trajectories of people with chronic life-limiting conditions other than cancer, and embed this adapted knowledge “upstream” into the delivery of care across care settings [57], requires consideration if improvements for the dying in acute care are to be achieved. Interventions such as using the “individualized” version of the CANHELP on medical teaching units is another promising improvement strategy to be considered if we are to achieve improvements in EOL care in these settings [64].

Despite the fact that family members were generally satisfied with the quality of care in the PCUs, findings suggest that there is room for improvement in areas that PCUs aim to excel. We might have expected to see higher satisfaction scores for all of the CANHELP subscales given the emphasis in palliative care on communication, decision making, symptom management and attention to psychosocial and spiritual concerns. Though illness management was the only subscale with a statistically significant difference in family member ratings between PCUs and ICUs, ICU ratings were observed to be higher for all but one subscale. Perhaps this is not surprising given that the ICU environment tends to have higher staffing ratios than most inpatient settings [65, 66]. At the same time, ICU is an environment that is highly technical, institutionalized, and where staff have not traditionally been exposed to much formal palliative care training [67, 68]. The lower than expected PCU scores run counterintuitive to clinical reports where families in palliative care report how deeply grateful they are for the care, services and support provided [31, 69]. However, it may be that our study of bereaved family members provides us with a different picture of their perceptions of care quality. Some reports suggest that family members are sometimes reluctant to be critical of palliative care services when the patient is alive because they do not want their complaints conceived as being non-appreciative of care and support or they do not want their critical comments to influence care that the patient receives [70, 71]. This is consistent with the work of the Picker Institute in the United Kingdom that has shown that real time satisfaction scores tend to be more favorable than retrospective scores and may lead to the conclusion that quality of care is better than it really is [72].

Finally, as Fig. 2 reveals, psychological variables and type of setting accounted for most of the explained variances in the CANHELP subscales and total score. That is, while psychological variables, mainly resilience of family members, play an important role in perceptions of the quality of EOL care, the type of care setting in which EOL care occurs, and several other characteristics of family members (e.g., their employment status, whether they provide care, and whether they live with the care recipient), are also important considerations in how bereaved family members appraise the quality of EOL care. These findings are not unexpected but point to some possible sites for targeted intervention. For example, acute care hospital interventions such as advance care planning has been shown to improve EOL care, enhance patient and family satisfaction and reduce stress, anxiety and depression among surviving relatives [73]. Likewise, research on resilience is being used to design interventions in other populations (i.e., helping families cope with a parent with a depressive disorder) and that may have applicability to supporting families in EOL care situations [74]. Focused attention on improving the delivery of high quality EOL care in all inpatient settings and on supporting families should be a goal for health system managers and administrators. The aging of the population along with increasing numbers of people diagnosed with chronic life-limiting illnesses will necessitate expansion or revision of existing services to meet the needs for EOL care, at least into the foreseeable future. Home care is often cited as the current solution to meet rising needs for palliative care [75, 76] and is reported to be the place where most people would prefer to be cared for and die [77–80]. However, without substantive investments in home-based palliative care it is unclear how this goal will be achieved or even sustained without adding significant cost to an already fragile system [75, 81] especially without shifting the financial burden to families [82]. Further, while findings not surprisingly suggest that resilient people are more likely to be satisfied with their care, there is a concurrent concern that resiliency may mask the very real quality of care and systemic issues that prevent excellent EOL care from occurring in inpatient settings. Research has shown that lack of knowledge of how to complain, low expectations, feelings of gratitude, fear of retribution and deference to health professionals may mask the problems patients and families face in receiving quality care [83–85]. Indeed, analyses of qualitative data collected for this study and published elsewhere [86] suggests that family members sometimes rationalize negative care experiences as an unavoidable reality within a constrained health care system, excusing front-line staff and the larger health care system from responsibility when undesirable care occurs.

### Limitations

Study findings should be considered in light of the fact that data are limited to one health region and the findings are undoubtedly influenced by the particular health service context and resource base available. In addition, there are small sample sizes within several of the settings though this is somewhat mitigated by a relatively good response rate among settings. Only English speaking people were surveyed and in a health region where English is the dominant language. Perceptions of care quality may differ in bereaved family membes with culturally and linguistically different backgrounds. Despite these limitations, the study raises some important questions in need of further exploration.

## Conclusion

Improving care at the EOL is a key policy direction to improve the quality of life for patients facing life-limiting conditions and their family members [87–89]. Understanding how quality of care is perceived by family members across inpatient care settings is one way to determine specific domains of care that are in need of improvement and to begin to address barriers to quality care. Although enhancing quality of EOL care is important in all settings, this study adds to the increasing body of evidence suggesting a critical need to focus on improvements in acute care, and suggests that care provided in palliative care units might also require attention.

## Abbreviations

ANCOVA: Analysis of covariance
ANOVA: Analysis of variance
CANHELP: Canadian health care evaluation project
ECU: Extended care unit
EOL: End of life
ICU: Intensive care unit
MCU: Medical care unit
PCU: Palliative care unit
TRIG: Texas revised inventory of grief
